# Potential Molecular Mechanisms of Ephedra Herb in the Treatment of Nephrotic Syndrome Based on Network Pharmacology and Molecular Docking

**DOI:** 10.1155/2022/9214589

**Published:** 2022-07-05

**Authors:** Tianwen Yao, Qingliang Wang, Shisheng Han, Yan Lu, Yanqiu Xu, Yi Wang

**Affiliations:** ^1^Department of Nephrology, Yueyang Hospital of Integrated Traditional Chinese and Western Medicine, Shanghai University of Traditional Chinese Medicine, Shanghai 200437, China; ^2^Department of Emergency, Yueyang Hospital of Integrated Traditional Chinese and Western Medicine, Shanghai University of Traditional Chinese Medicine, Shanghai 200437, China

## Abstract

**Objective:**

To explore the possible mechanisms of Ephedra herb (EH) in the treatment of nephrotic syndrome (NS) by using network pharmacology and molecular docking in this study.

**Methods:**

Active ingredients and related targets of EH were obtained from the Traditional Chinese Medicine Systems Pharmacology (TCMSP) database, and the gene names corresponding to the proteins were found through the UniProt database. Then, target genes related to NS were screened out from GeneCards, PharmGKB, and OMIM databases. Next, the intersection targets were obtained successfully through Venn diagram, which were also seen as key target genes of EH and NS. Cytoscape 3.9.0 software was used to construct the effective “active ingredient-target” network diagram, and “drug-ingredient-target-disease (D-I-T-D)” network diagram. After that, the STRING database was used to construct a protein-protein interaction (PPI) network. Furthermore, Gene Ontology (GO) and Kyoto Encyclopedia of Genes and Genomes (KEGG) pathway enrichment involved in the targets were performed by the DAVID database and ClueGO plugin in Cytoscape. Finally, AutoDockTools software was used for molecular docking to verify the binding strength between main active ingredients and key target proteins.

**Results:**

A total of 22 main active ingredients such as quercetin, kaempferol, luteolin, and naringenin were obtained, which could act on 105 targets related to NS. Through PPI network, 53 core targets such as AKT1, TNF, IL6, VEGFA, and IL1B were found, which might play a crucial role in the treatment of NS. Meanwhile, these targets were significantly involved in PI3K-Akt signaling pathway, TNF signaling pathway, AGE-RAGE signaling pathway, hepatitis B, and pathways in cancer through GO and KEGG enrichment analysis. The docking results indicated that active ingredients such as kaempferol, luteolin, quercetin, and naringenin all had good binding to the target protein AKT1 or TNF. Among them, luteolin and naringenin binding with AKT1 showed the best binding energy (-6.2 kcal/mol).

**Conclusion:**

This study indicated that the potential mechanism of EH in treating NS may be related to PI3K-Akt signaling pathway, TNF signaling pathway, and AGE-RAGE signaling pathway, which provided better approaches for exploring the mechanism in treating NS and new ideas for further in vivo and in vitro experimental verifications.

## 1. Introduction

Nephrotic syndrome (NS) is characterized by massive proteinuria (greater than 3.5 g per day), hypoalbuminemia (less than 30 g/L), hyperlipidemia, systemic edema, and various complications [1]. As one of the most frequent glomerular diseases affecting more than millions of people worldwide, NS is caused by the dysfunctional glomerular filtration barrier [2]. In the last decades, NS has become a prevalent disease associated with high morbidity in 20%-40% of children [3, 4]. Nowadays, steroids and immunosuppressive drugs are the most common methods in the treatment of NS at home and abroad [5]. Usually, NS responds well to steroids [6]. However, frequent recurrences are common, which will require multidrug therapy with long-term side effects [7]. Obviously, those problems not only increase the difficulty of treatment but also accelerate the development of end-stage renal disease (ESRD), which can be a catastrophic event for the patient, family, and even society [8]. Therefore, it is crucial to search more advanced and natural drugs with less side effects for the treatment of NS.

Ephedra herb (EH), also named as mahuang in Chinese, belongs to the stems of *Ephedra sinica* Stapf, *Ephedra intermedia* Schrenk & C.A.Mey., and *Ephedra equisetina* Bunge [9]. EH is one of the most ancient herbs recorded in Sheng Nong's Herbal Classic, which plays an important role in terms of relieving exterior syndrome by diaphoresis, as well as inducing diuresis to alleviate edema. Chemical analyses demonstrate that a variety of specific ingredients have been extracted from EH, including flavonoids, alkaloids, organic acids, polysaccharides, tannins, volatile oils, and other active ingredients [10]. EH has been used for medicinal purposes for thousands of years with its biological and pharmacological properties, such as antioxidative, antimicrobial, anti-inflammatory, antiallergic, and antiproliferative effects [11–13]. Recently, it is widely used to treat bronchial asthma, cardiovascular related diseases, immune system diseases, cancer, and other diseases [14–16]. Moreover, it is reported that EH has positive effects on the treatment of NS through reducing protein in the urine, alleviating kidney functional and structural impairment [17, 18]. However, the mechanism of EH to treat NS is unclear, and the targets and pathways of its effective chemical ingredients are still unknown, which need to be further studied.

As a new technology to analyze the potential mechanism of compounds or single herbs, network pharmacology has been widely used in the field of TCM. It is known that network pharmacology is an emerging discipline that designs drugs based on systems biology theory and biological system network analysis [19]. So, network pharmacology is aimed at clarifying the molecular mechanism of drugs, providing guidance for the prediction of TCM, and exploring the relationships between drugs and diseases from a macro perspective [20], which is especially suitable for conducting multicomponent, multitarget, and synergistic studies of TCM [21]. Molecular docking is a computational method that can be used to study the binding interaction between molecule ligands and target proteins [22]. This helps not only in understanding the mechanism of their biological action but also accelerating the process of drug discovery [23].

**Figure 1:**
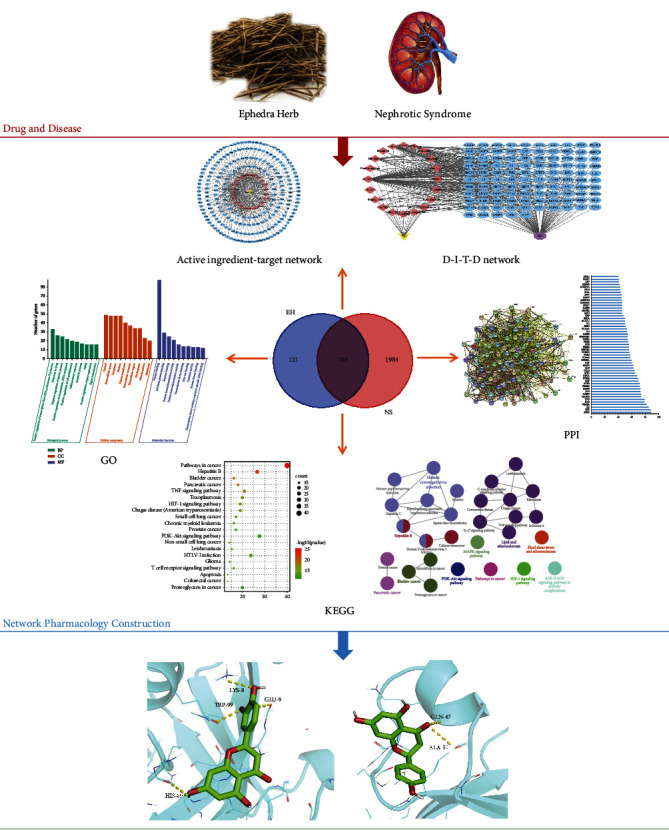
A comprehensive strategy diagram for the study of the mechanism of EH acting on NS.

In this study, network pharmacology and molecular docking methods were both used to screen the possible targets of EH intervention in NS and establish the network of EH active ingredients-targets-pathways-diseases, which may provide a basis for relevant experimental researches (Figure 1).

## 2. Materials and Methods

### 2.1. Screening Active Ingredients of EH

Traditional Chinese Medicine Systems Pharmacology (TCMSP, http://tcmspw.com/tcmspsearch.php) database is an open and accessible database resource [24]. Furthermore, it acts as a unique systems pharmacology platform of Chinese herbal medicines to explore the relationships between drugs, targets, and diseases. The keyword “Mahuang” was inputted into the search box to retrieve all the active ingredient data of EH. Because oral bioavailability (OB) and drug-likeness (DL) are important evaluation indexes for drugs to participate in the process of ADME (absorption, distribution, metabolism, and excretion), we took OB and DL as screening conditions [25]. In order to obtain active ingredients of EH more comprehensively, we set the condition of OB ≥ 30% and DL ≥ 0.18 to select them [26].

### 2.2. Screening Active Ingredient Targets of EH

We took “mahuang” as the keyword and searched “Related Targets” in the TCMSP database to find target proteins corresponding to the active ingredients of EH [27]. Then, the gene names corresponding to those target proteins were specified through UniProt (https://www.uniprot.org/) with *Homo sapiens* as the selected species. The Cytoscape software (version 3.9.0) was used to construct a network between active ingredients of EH and their corresponding target points. In the network, each node represented a target gene or molecule, and the connecting line between two nodes represented the relationship between them. It meant the stronger the degree of the node, the greater the role of the target in the network [28].

### 2.3. Collection of Target Genes Related to NS

With the keyword of “nephrotic syndrome”, target genes related to NS were collected from GeneCards (https://www.genecards.org), PharmGKB (https://www.pharmgkb.org/), and OMIM (https://www.omim.org) databases. The three databases are recognized as good references for the collection of disease targets. Results accessed from GeneCards were screened for the relevance score ≥ 1.00 as the screening index. The repeated target genes corresponding to NS and the active ingredients of EH were deleted by Excel. Finally, the intersection targets were obtained successfully through Venn diagram in the website http://bioinformatics.psb.ugent.be/webtools/Venn/.

### 2.4. Construction of Drug-Ingredient-Target-Disease (D-I-T-D) Network

We obtained intersection target genes of EH and NS by Venn diagram. Then, a visual comprehensive network (D-I-T-D) was built by Cytoscape software (version 3.9.0) based on the interaction between drug (EH), active ingredients, intersection target genes, and diseases (NS).

### 2.5. Construction of Protein-Protein Interaction Network

The STRING database (https://string-db.org/) is an online protein-protein interaction (PPI) analysis database. It can collect and integrate data about known and predicted protein-protein associations from many organisms, including both direct and indirect interactions [29]. Therefore, the intersection targets of EH and NS were inputted into the STRING database to construct the PPI network. In order to ensure the high confidence of information, the minimum interaction score was set as 0.4, the species was set as *Homo sapiens*, and the isolated proteins were hidden.

### 2.6. Gene Ontology (GO) and Kyoto Encyclopedia of Genes and Genomes (KEGG) Pathway Enrichment

To further explore the pathways of the disease, the intersection targets of EH and NS were entered in the DAVID database (https://david.ncifcrf.gov/).

It is an online biological knowledge base and analytic tool, which is mainly used to obtain various biological information, including gene functional classification, functional annotation, and enriched pathways [30]. GO enrichment analysis is composed of biological process (BP), cellular component (CC), and molecular function (MF). KEGG is a knowledge base used to describe molecular interactions and reveal known metabolic pathways. The species was defined as *Homo sapiens* to perform GO and KEGG pathway enrichment analysis. According to the gene number and *P* value, the top 10 GO and KEGG items were selected for further analysis, respectively. Meanwhile, KEGG pathway enrichment analysis was also performed using ClueGO plugin in Cytoscape.

### 2.7. Molecular Docking of Active Ingredients with Key Targets

We selected two targets with the largest value in the PPI network as the receptors and chose the corresponding active ingredients as the ligands for molecular docking verification. Firstly, the PubChem database (https://pubchem.ncbi.nlm.nih.gov) was used to obtain the 2D structure diagrams of active ingredients, and we transformed them into 3D structure diagrams through Chem3D software (version 14.0). Then, protein crystal forms corresponding to the targets were obtained from the PDB database (https://www.rcsb.org/). After that, we imported them into PyMOL software (version 2.5) to remove water molecules. Next, the ingredient and corresponding protein were both processed by AutoDockTools 1.5.7 software and saved in pdbqt format as a ligand and a receptor, respectively. Finally, docking work was performed to analyze the results using PyMOL software and AutoDock Vina software (version 1.1.2).

## 3. Results

### 3.1. Screening Active Ingredients of EH

We used “mahuang” as the keyword to search active ingredients in the TCMSP database and found 363 kinds of chemical ingredients. With the screening conditions of OB ≥ 30% and DL ≥ 0.18, a total of 23 potential active ingredients satisfying the conditions were obtained in Table 1. Then, we screened out ingredients without corresponding targets and confirmed 22 potential active ingredients of EH, respectively: leucopelargonidin, herbacetin, resivit, kaempferol, delphinidin, quercetin, luteolin, beta-sitosterol, stigmasterol, (+)-catechin, mandenol, 24-ethylcholest-4-en-3-one, poriferast-5-en-3beta-ol, diosmetin, naringenin, taxifolin, campest-5-en-3beta-ol, eriodictyol, genkwanin, pectolinarigenin, (+)-leucocyanidin, and truflex OBP. They were the main active ingredients of EH in the treatment of NS. Finally, their chemical abstracts service (CAS) number, chemical structure, molecular formula, and molecular weight are shown in Table 2.

### 3.2. Screening Active Ingredient Targets of EH and Construction of Active Ingredient-Target Network

**Figure 2:**
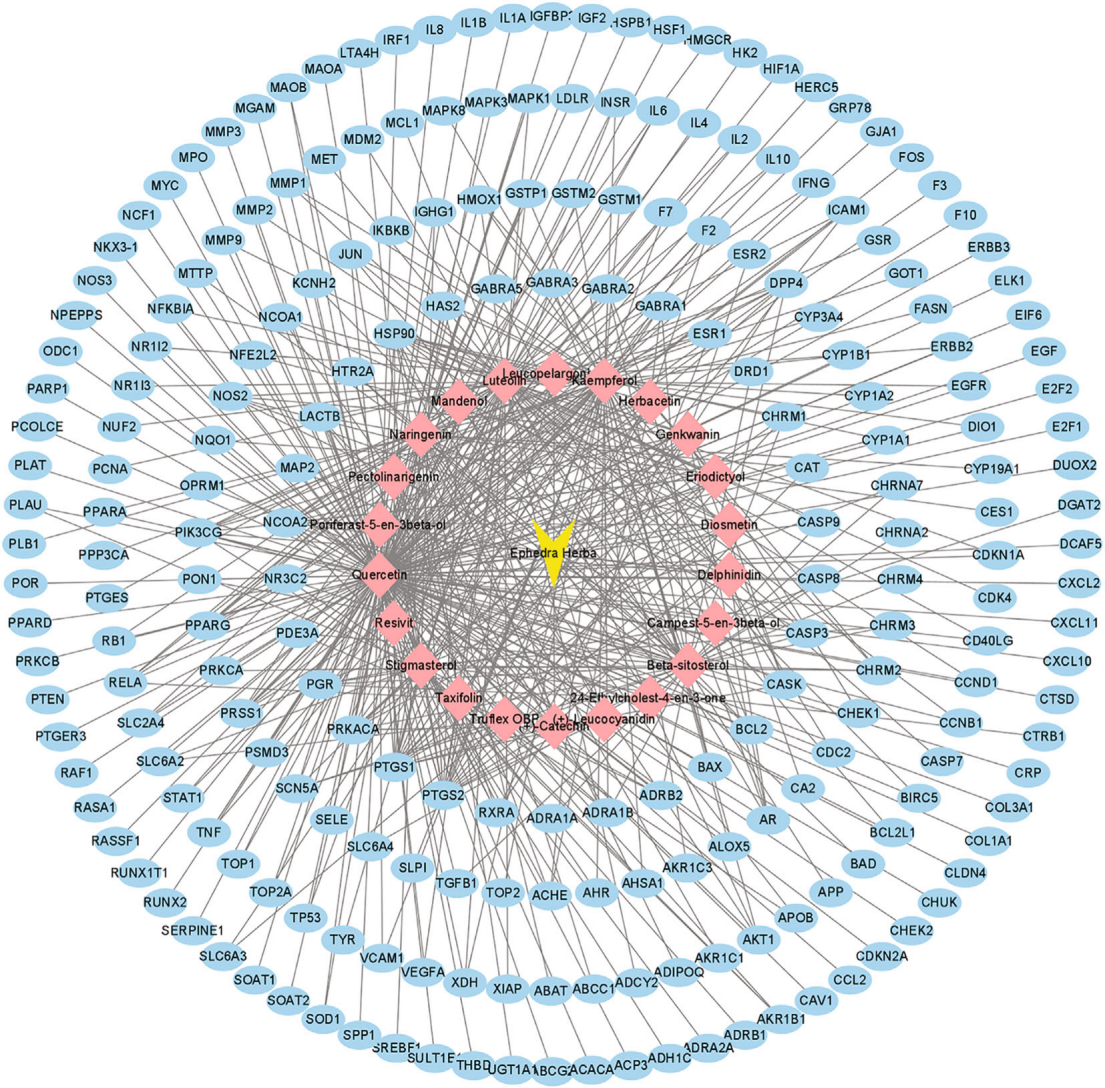
Active ingredient-target network of EH.

22 effective chemical ingredients of EH were inputted into the TCMSP platform, and 226 corresponding proteins were obtained. Then, target gene names corresponding to those proteins were found by UniProt. Finally, the active ingredients and targets are imported into Cytoscape 3.9.0 software to construct active ingredient-target network. As shown in Figure 2, the network between EH active ingredients and corresponding targets consisted of 249 nodes (1 herbal medicine node, 22 active ingredient nodes, and 226 corresponding target nodes) and 515 edges. The yellow inverted triangle node represented EH, the pink diamond nodes represented the active ingredients of EH, and the blue oval nodes represented the target genes. It was obvious that quercetin has the most number of targets. It could also be concluded from the figure that each active ingredient of EH had multiple targets, and each target could correspond to multiple active ingredients, which deeply showed the characteristics of multicomponent and multitarget actions of EH.

### 3.3. Collection of Gene Targets Related to NS

**Figure 3:**
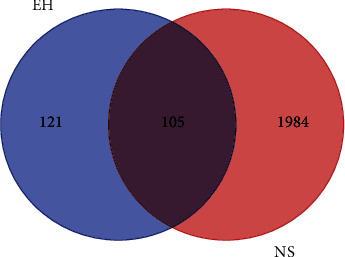
Intersection targets of EH and NS.

The keyword “nephrotic syndrome” was used to search relevant genes in GeneCards, PharmGKB, and OMIM databases. Then, a total of 2089 genes were obtained after deletion. Finally, intersection targets were acquired by overlapping the above targets of EH and NS via the Venn diagram. As shown in Figure 3, there were 105 intersection targets of EH and NS.

### 3.4. Construction of Drug-Ingredient-Target-Disease (D-I-T-D) Network

**Figure 4:**
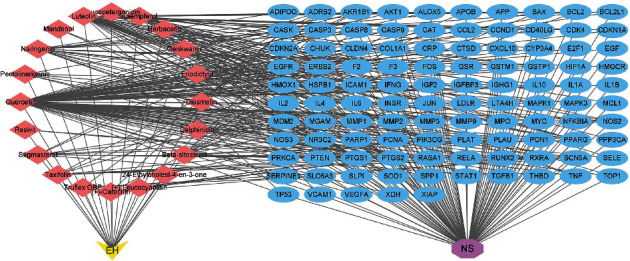
Drug-ingredient-target-disease (D-I-T-D) network.

The drug (EH), active ingredients, target genes, and disease (NS) were imported into Cytoscape 3.9.0 software to construct the D-I-T-D network. As shown in Figure 4, the yellow inverted triangle node represented EH, the purple octagon node represented NS, the pink diamond nodes represented the active ingredients of EH, and the 105 blue oval nodes represented the overlapping genes between disease and drug. It could be seen from the figure that quercetin had the most number of overlapping genes. Furthermore, it clearly showed how EH treated NS through active ingredients and target genes.

### 3.5. Construction of Protein-Protein Interaction Network

**Figure 5:**
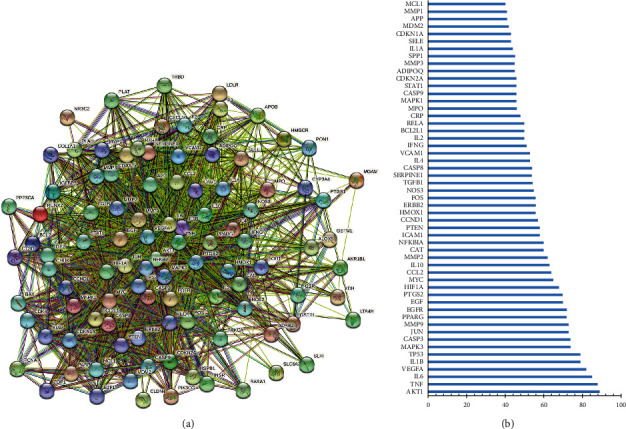
(a) PPI network diagram of the intersection targets of EH and NS. (b) 53 core targets arranged according to degree value.

We imported 105 overlapping targets into the STRING database to obtain the PPI relationship. As shown in Figure 5(a), the network had a total of 104 nodes and 2064 edges after removing the free one. In addition, the mean degree value of those overlapping targets was 39.70. According to the degree value, they were ranked from high to low. As shown in Figure 5(b), we selected targets with value greater than 39.70 and finally obtained 53 core targets. In terms of degree value, the top 10 key target proteins were RAC-alpha serine/threonine-protein kinase (AKT1), tumor necrosis factor (TNF), interleukin 6 (IL6), vascular endothelial growth factor a (VEGFA), interleukin 1 beta (IL1B), tumor protein P53 (TP53), mitogen-activated protein kinase 3 (MAPK3), caspase 3 (CASP3), jun proto-oncogene, AP-1 transcription factor subunit (JUN), and matrix metallopeptidase 9 (MMP9). It was believed that these targets may be significant for EH in the treatment of NS.

### 3.6. GO and KEGG Pathway Enrichment

**Figure 6:**
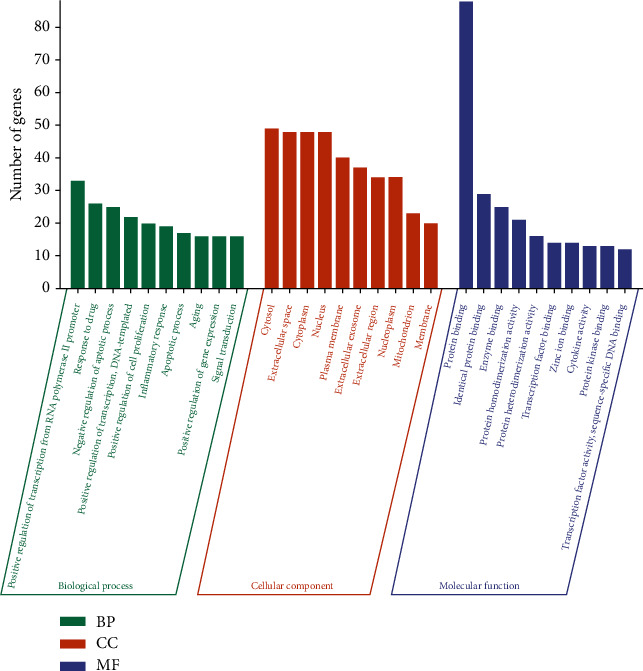
GO biological function analysis of intersection target genes.

To further explore the mechanism of EH on NS more systematically, the intersection target genes were inputted into DAVID database for GO analysis and KEGG pathway analysis (*P* < 0.01). It showed that the predicted target genes of EH were mainly enriched in 540 biological processes (BP), 64 cellular components (CC), and 93 molecular functions (MF). According to the number of target gens, the top 10 enriched conditions of GO analysis were listed, respectively, in Figure 6. It could be seen that green represented BP, orange represented CC, and purple represented MF. BP mainly included positive regulation of transcription from RNA polymerase II promoter, response to drug, negative regulation of apoptotic process, positive regulation of transcription, DNA-templated, positive regulation of cell proliferation, inflammatory response, apoptotic process, aging, positive regulation of gene expression, and signal transduction. CC mainly included cytosol, extracellular space, cytoplasm, nucleus, plasma membrane, extracellular exosome, extracellular region, nucleoplasm, mitochondrion, and membrane. MF mainly included protein binding, identical protein binding, enzyme binding, protein homodimerization activity, protein heterodimerization activity, transcription factor binding, zinc ion binding, cytokine activity, protein kinase binding, transcription factor activity, and sequence-specific DNA binding.

**Figure 7:**
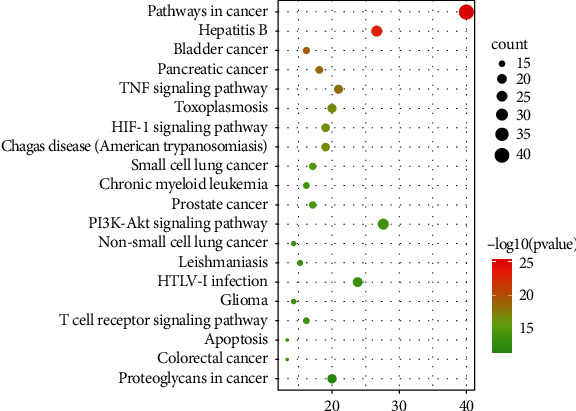
KEGG pathway analysis of intersection target genes through DAVID database.

**Figure 8:**
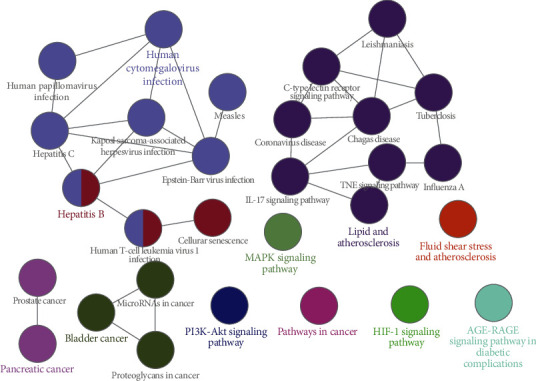
KEGG pathway analysis of intersection target genes through ClueGO.

The KEGG pathway enrichment was, respectively, completed via DAVID database and ClueGO plugin in Cytoscape. Through DAVID database, the KEGG pathway enrichment obtained a total of 114 pathways, of which the top 20 pathways were shown according to the size of the *P* value in Figure 7. The *y*-axis represented the name of the pathway, and the *x*-axis represented the ratio of target genes to background genes. Besides, the size of dot represented the count of genes on different pathways, and the color of dot represented the significance of enrichment. Obviously, the data showed that EH mainly regulated pathways in cancer, hepatitis B, PI3K-Akt signaling pathway, HTLV-I infection, and TNF signaling pathway to treat NS. Through ClueGO plugin in Cytoscape, we obtained 29 representative pathways (*P* < 0.01) as shown in Figure 8. These pathways were divided into 11 categories based on signaling pathway functional clustering analysis, including AGE-RAGE signaling pathway in diabetic complications, PI3K-Akt signaling pathway, pathways in cancer, HIF-1 signaling pathway, hepatitis B, MAPK signaling pathway, lipid and atherosclerosis, fluid shear stress and atherosclerosis, bladder cancer, pancreatic cancer, and human cytomegalovirus infection.

### 3.7. Molecular Docking of Active Ingredients with Key Targets

**Figure 9:**
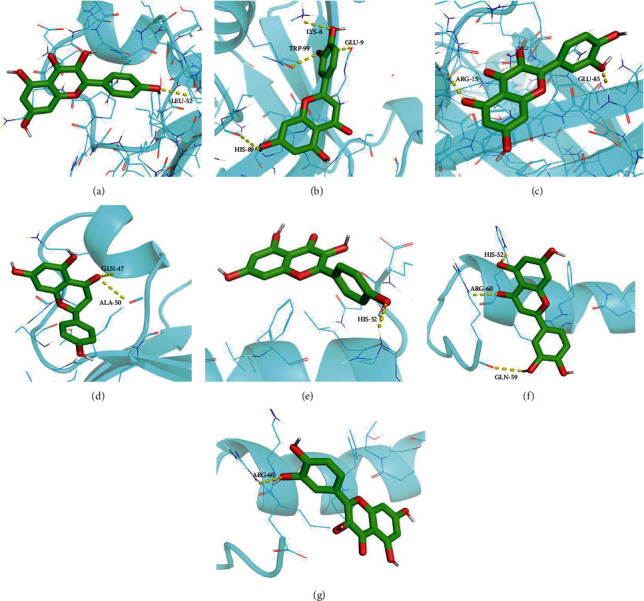
Molecular docking of active ingredients with key targets: (a) kaempferol with AKT1, (b) luteolin with AKT1, (c) quercetin with AKT1, (d) naringenin with AKT1, (e) kaempferol with TNF, (f) luteolin with TNF, and (g) quercetin with TNF. The molecule was represented in a ball-stick model with atoms C and O in green and red, respectively.

We selected AKT1 and TNF as the protein receptors because they had the highest degree value in the PPI network. Accordingly, the active ingredients including kaempferol, luteolin, quercetin, and naringenin were used as ligands for molecular docking verification. As shown in Table 3, the affinities between these ingredients and targets were lower than -5.0 kcal/mol, which demonstrated that the main active ingredients of EH had a strong binding activity with targets. According to the docking results, the binding energy of kaempferol to AKT1 and TNF was -6.1 and -5.1 kcal/mol, the binding energy of luteolin to AKT1 and TNF was -6.2 and -5.3 kcal/mol, the binding energy of quercetin to AKT1 and TNF was -5.7 and -5.0 kcal/mol, and the binding energy of naringenin to AKT1 was -6.2 kcal/mol. Among them, luteolin and naringenin binding with AKT1 showed the best binding energy (-6.2 kcal/mol). Besides, four amino acid residues in AKT1, LYS-8, TRP-99, GLU-9, and HIS-89, formed multiple binding locations with luteolin (Figure 9(b)), while LEU-52 also bound to kaempferol through hydrogen bonding (Figure 9(a)). Two amino acid residues in AKT1, ARG-15 and GLU-85, formed multiple hydrogen bonds with quercetin (Figure 9(c)), and another two amino acid residues, GLN-47 and ALA-50, formed hydrogen bonds with naringenin (Figure 9(d)). Three amino acid residues in TNF, HIS-52, ARG-60, and GLN-59, formed multiple binding locations with luteolin (Figure 9(f)), while HIS-52 and ARG-60 also bound to kaempferol and quercetin, respectively, through hydrogen bonding (Figures 9(e) and 9(g)).

## 4. Discussion

NS is a disease with considerable morbidity and high risk for relapse [31]. Despite developed medical conditions and increasingly diverse ways of reducing proteinuria, NS still cannot be effectively treated [32]. As a famous Chinese herbal medicine with a long history, EH has a wide range of pharmacological effects in the treatment of bronchial asthma, immune system diseases, cardiovascular related diseases, and cancer [33, 34]. Recently, EH is frequently used alone or in conjunction with other herbs to treat NS, but the relevant mechanism is not yet clear.

As is known, network pharmacology is a rapidly emerging discipline, which has the same thinking with TCM. In this study, we used network pharmacology theory and related tools to select the active ingredients and related targets of EH and linked them with NS. A total of 23 active ingredients with OB ≥ 30% and DL ≥ 0.18 were screened in the TCMSP database. From the “active ingredient-target” network diagram and “drug-ingredient-target-disease” network diagram, it could be seen that active ingredients including quercetin, kaempferol, luteolin, and naringenin were important nodes in the network. Besides, the network also intuitively displayed the specific relationship between EH and NS through 105 consensus genes.

Among them, quercetin is a natural flavonoid extracted from EH, other herbs, and numerous fruits and vegetables, which is known as a powerful herbal antioxidant. Pharmacological studies have shown that quercetin has several biological activities, such as antioxidant, anti-inflammatory, and anticarcinogenic effects [35]. More and more studies have proved that quercetin exerts renal protective effects through a variety of ways, including inhibiting ferroptosis, preventing the aging of the kidney cells, alleviating renal fibrosis and apoptosis, and repressing oxidative stress and inflammation [36, 37]. As a natural anti-inflammatory compound, kaempferol has been reported to exert curative effects on kidney inflammatory damage [38]. Recently, numerous studies have shown that kaempferol has salutary effects for anti-inflammatory, antioxidant, analgesic, and anticancer properties [39]. With those properties, kaempferol has been demonstrated to exert beneficial therapeutic applications in nephrolithiasis, edema, and hyperlipidemia [40, 41]. Luteolin is a nontoxic flavonoid, which has become a hot area of research in an alternative medicine for several years [42]. Numerous studies have confirmed that luteolin has a wide range of biological activities, including antiapoptosis, antioxidative, anti-inflammatory, and anticancer properties [43]. It is generally accepted that luteolin exerts therapeutic effects on NS and other kidney injury [44, 45]. Therefore, it is speculated that these active ingredients may be important material basis of EH to treat NS.

The PPI network of the intersection targets of EH and NS clearly showed that AKT1, TNF, IL6, VEGFA, IL1B, TP53, MAPK3, CASP3, JUN, and MMP9 were the top 10 nodes with the highest degree value, which interacted with multiple active ingredients and played a key role in the network diagram. AKT family exists in three different isoforms, and they are named AKT1, AKT2, and AKT3, respectively. These unique isoforms are differentially expressed in mangy signaling pathways according to the developmental stage. Among them, AKT1 is involved in the proliferation, activation, apoptosis, and cell survival of interstitial fibroblasts and glomerular mesangial cells [46]. Kim et al. found that the deletion of AKT1 resulted in the attenuation of renal fibrosis and tubular dedifferentiation, which proved that AKT1 might serve as a therapeutic target in AKI-to-CKD progression [47]. Lin et al. reported that uncoupled mitochondrial respiration and increased oxidative stress were both found when mitochondria AKT1 was inhibited, which suggested that AKT1 signaling could be a novel target to develop new strategies for better treatment of various kidney diseases [48]. TNF is primarily produced by mononuclear macrophages and can prevent the body from the infringement by killing tumor cells. It is also proved to be an important cytokine with a wide range of biological effects, which is connected with the susceptibility and development of immune diseases, including NS [49].

GO analysis can be used to make a significant annotation of gene products. Among them, BP enrichment showed the coupling effect and transport mode of proteins in biological pathways. The results of CC analysis proved that intersection proteins participated in the cellular environment. Through MF analysis, we could demonstrate that some protein receptor activities were regulated by drugs, such as protein binding, which was closely related to proteinuria [50]. KEGG pathway enrichment analysis guides us to combine genomes with cellular and species to figure out the potential pathways of EH against NS. Our results showed that EH could be applied in the treatment of NS through PI3K-Akt signaling pathway, TNF signaling pathway, AGE-RAGE signaling pathway, hepatitis B, and pathways in cancer. In those pathways, multiple targets such as AKT1 and TNF were simultaneously regulated by active ingredients such as quercetin, kaempferol, and luteolin.

The PI3K-Akt signaling pathway has been proposed to modulate diverse biological processes and plays a key role in podocyte apoptosis, which is connected with the progress of proteinuria and renal function [51]. It has been reported that EH exerts anti-inflammatory and antioxidative stress effects through promoting the phosphorylation of PI3K and AKT proteins [52]. As the main component of EH, quercetin has been proved to improve renal fibrosis and apoptosis in chronic renal failure rats, as well as reducing water retention and toxin accumulation by inhibiting the PI3k/Akt pathway through targeting PIK3R [53]. Thus, regulating the PI3K/AKT pathway in podocytes through EH may be an important potential targeted therapy for NS in the future [54]. As one of important inflammatory pathways, the TNF signaling pathway plays a central role in the regulation of different cellular events such as immunity, apoptosis, cell proliferation, and differentiation [55]. It has been proved that the TNF signaling pathway is connected with cholesterol-dependent podocyte apoptosis and albuminuria, which may be primarily activated immediately in the process of various NS progression, including diabetic nephropathy and focal segmental glomerulosclerosis [56, 57]. It has been demonstrated that EH exhibits antioxidant, anti-inflammatory, and antiarthritic properties by decreasing proinflammatory cytokines, including inhibiting the TNF signaling pathway [58]. Besides, EH has been proved to exert anti-inflammatory potential via downregulation of TNF-*α* [59]. As one of the active ingredients extracted from EH, kaempferol can reduce the intensity of inflammatory processes by inhibiting the secretion of proinflammatory cytokine TNF-*α* and additionally increasing the expression of anti-inflammatory cytokine IL-10 [60]. Furthermore, the protective effect of kaempferol against streptozotocin-induced diabetic nephropathy has been proved to be associated with the downregulation of TNF-*α* and IL-6 [61]. The AGE-RAGE signaling pathway is connected with the regulation of oxidative stress and inflammation. When it is activated, nicotinamide adenine dinucleotide phosphate oxidase-induced reactive oxygen is produced, which will give rise to oxidative stress [62]. Therefore, targeting at the AGE-RAGE signaling pathway could be the promising potential approach for improving NS and ageing kidney.

The results of molecular docking were consistent with the results of network pharmacology because active ingredients such as kaempferol, luteolin, quercetin, and naringenin all had good binding to the target protein AKT1 or TNF, which provided a strong basis for the results of network pharmacology. So, the feasibility of TCM such as EH can be affirmed in the treatment of NS.

## 5. Conclusions

On the basis of network pharmacology and molecular docking, this study focused on the active ingredients of EH and its multitarget and multipathway characteristics in the process of treating NS. The results indicated that the potential mechanism of EH in treating NS may be related to PI3K-Akt signaling pathway, TNF signaling pathway, and AGE-RAGE signaling pathway. It may provide new ideas and better approaches for the treatment of NS. However, further in vivo and in vitro experimental verifications are expected to be conducted in the future.

## Figures and Tables

**Table 1:** Potential active ingredients of EH.

Number	Mol ID	Molecule name	OB (%)	DL
1	MOL010788	Leucopelargonidin	57.97	0.24
2	MOL002823	Herbacetin	36.07	0.27
3	MOL010489	Resivit	30.84	0.27
4	MOL000422	Kaempferol	41.88	0.24
5	MOL004798	Delphinidin	40.63	0.28
6	MOL000098	Quercetin	46.43	0.28
7	MOL000006	Luteolin	36.16	0.25
8	MOL000358	Beta-sitosterol	36.91	0.75
9	MOL000449	Stigmasterol	43.83	0.76
10	MOL000492	(+)-Catechin	54.83	0.24
11	MOL001494	Mandenol	42.00	0.19
12	MOL001506	Supraene	33.55	0.42
13	MOL001755	24-Ethylcholest-4-en-3-one	36.08	0.76
14	MOL001771	Poriferast-5-en-3beta-ol	36.91	0.75
15	MOL002881	Diosmetin	31.14	0.27
16	MOL004328	Naringenin	59.29	0.21
17	MOL004576	Taxifolin	57.84	0.27
18	MOL005043	Campest-5-en-3beta-ol	37.58	0.71
19	MOL005190	Eriodictyol	71.79	0.24
20	MOL005573	Genkwanin	37.13	0.24
21	MOL005842	Pectolinarigenin	41.17	0.30
22	MOL007214	(+)-Leucocyanidin	37.61	0.27
23	MOL011319	Truflex OBP	43.74	0.24

**Table 2:** Information table of active ingredients of EH.

CAS number	Ingredient name	Chemical structure	Molecular formula	Molecular weight
520-17-2	Leucopelargonidin	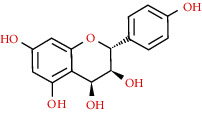	C_15_H_14_O_6_	290.27
527-95-7	Herbacetin	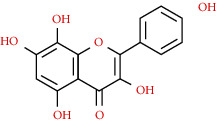	C_15_H_10_O_7_	302.25
480-17-1	Resivit	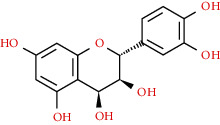	C_15_H_14_O_7_	306.27
520-18-3	Kaempferol	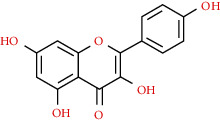	C_15_H_10_O_6_	286.24
13270-61-6	Delphinidin	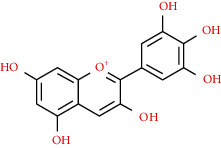	C_15_H_11_O_7_	303.24
117-39-5	Quercetin	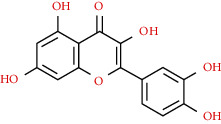	C_15_H_10_O_7_	302.24
491-70-3	Luteolin	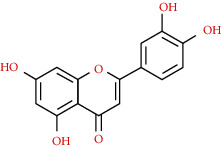	C_15_H_10_O_6_	286.24
83-46-5	Beta-sitosterol	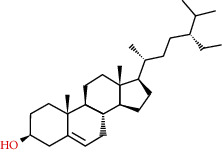	C_29_H_50_O	414.71
CAS number	Ingredient name	Chemical structure	Molecular formula	Molecular weight
83-48-7	Stigmasterol	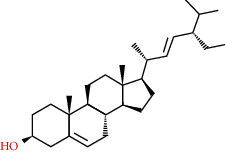	C_29_H_48_O	412.69
154-23-4	(+)-Catechin	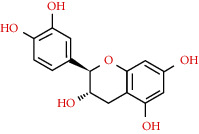	C_15_H_14_O_6_	290.27
544-35-4	Mandenol	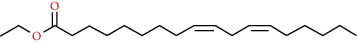	C_20_H_36_O_2_	308.50
67392-96-5	24-Ethylcholest-4-en-3-one	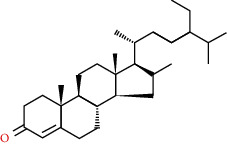	C_29_H_48_O	412.69
18525-35-4	Poriferast-5-en-3beta-ol	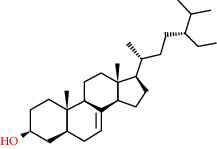	C_29_H_50_O	414.71
520-34-3	Diosmetin	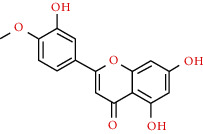	C_16_H_12_O_6_	300.26
480-41-1	Naringenin	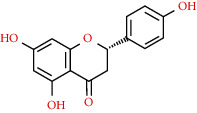	C_15_H_12_O_5_	272.25
480-18-2	Taxifolin	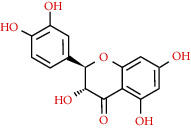	C_15_H_12_O_7_	304.25
CAS number	Ingredient name	Chemical structure	Molecular formula	Molecular weight
474-62-4	Campest-5-en-3beta-ol	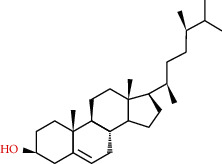	C_28_H_48_O	400.68
552-58-9	Eriodictyol	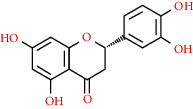	C_15_H_12_O_6_	288.25
437-64-9	Genkwanin	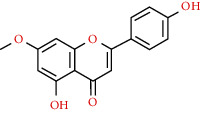	C_16_H_12_O_5_	284.26
520-12-7	Pectolinarigenin	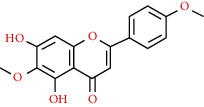	C_17_H_14_O_6_	314.29
69256-15-1	(+)-Leucocyanidin	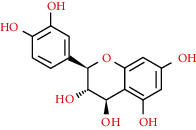	C_15_H_14_O_7_	306.29
84-78-6	Truflex OBP	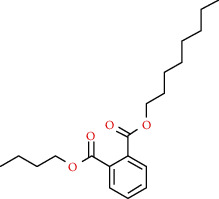	C_20_H_30_O_4_	334.45

**Table 3:** Molecular docking scores of major active ingredients and targets.

Number	Ingredient	Target	Affinity value (kcal/mol)
1	Kaempferol	AKT1	-6.1
2	Luteolin	AKT1	-6.2
3	Quercetin	AKT1	-5.7
4	Naringenin	AKT1	-6.2
5	Kaempferol	TNF	-5.1
6	Luteolin	TNF	-5.3
7	Quercetin	TNF	-5.0

## Data Availability

The data used to support the findings of this study are included within the article.
